# Are decorin gene variants associated with anterior cruciate ligament rupture susceptibility?

**DOI:** 10.5114/biolsport.2025.152112

**Published:** 2025-07-16

**Authors:** Kinga Łosińska, Agata Rzeszutko-Bełzowska, Krzysztof Ficek, Alison V. September

**Affiliations:** 1 Department of Physical Education, Gdansk University of Physical Education and Sport, Poland; 2 Faculty of Physical Education, University of Rzeszow, Poland; 3 Department of Sports Training, Institute of Sport Sciences, The Jerzy Kukuczka Academy of Physical Education, 40-065 Katowice, Poland; 4 Health Through Physical Activity, Lifestyle and Sport Research Centre (HPALS), Division of PhysiologicalSciences, Department of Human Biology, University of Cape Town, Rondebosch,Cape Town 7700, South Africa

**Keywords:** DCN, Case-control study, Polymorphism, ACL rupture, ACL injury

## Abstract

This study aimed to investigate whether two DCN gene single nucleotide polymorphisms (SNPs), rs13312816 (T > A) and rs516115 (A > G), are associated with the risk and severity of anterior cruciate ligament (ACL) injury. A total of 296 physically active, unrelated Caucasian males participated: 160 with noncontact ACL injuries and 136 healthy controls. Genotyping was conducted using TaqMan assays. Logistic regression and haplotype-based analyses were performed, adjusting for age and body mass. The minor A allele of rs13312816 was significantly more frequent in ACL cases than in controls (8.54% vs. 2.94%, P = 0.0047; OR = 3.08, 95% CI: 1.33–7.98). Individuals with the A/T genotype had higher odds of injury compared to T/T carriers (P_adj_ = 0.008; OR = 3.3, 95% CI: 1.44–7.53). No associations were found for rs516115 in the case– control comparison. Haplotype analysis showed that individuals with the [A;G] haplotype had increased odds of ACL injury (P_adj_ = 0.0095; OR = 3.29, 95% CI: 1.44–7.52). Within the injured group, rs13312816 A/T genotype was associated with multiple injuries (ACLF) (P_adj_ = 0.010; OR = 3.19, 95% CI: 1.36–7.48). For rs516115, both A/G (P_adj_j < 0.0001; OR = 6.03, 95% CI: 2.83–12.83) and G/G genotypes (P_adj_ < 0.0001; OR = 9.71, 95% CI: 2.57–36.77) were linked to ACLF. Haplotype analysis confirmed increased odds for multiple injuries in carriers of [A;G] (P_adj_ = 0.0099; OR = 3.34, 95% CI: 1.33–8.35) and [T;G] haplotypes (P_adj_ < 0.0001; OR = 4.79, 95% CI: 2.35–9.79). These findings suggest that DCN genetic variants, especially rs13312816 and specific haplotypes, contribute to ACL injury susceptibility and recurrence.

## INTRODUCTION

Anterior cruciate ligament (ACL) injuries represent a significant clinical and economic burden due to their high prevalence among athletes and the general population, as well as their long-term consequences, which include joint instability, post-traumatic osteoarthritis, and a decreased quality of life [1–5]. ACL injuries are commonly caused by non-contact mechanisms, such as sudden changes in direction, landing improperly from a jump, or rapid deceleration during physical activity. Despite advancements in preventive measures and surgical repair techniques, the incidence of ACL injuries remains high, particularly in sports involving rapid movements and pivoting [6, 7].

The pathophysiology of ACL injuries is multifactorial, with both environmental and genetic factors playing a significant role in injury susceptibility [8, 9]. Recent studies have highlighted the importance of genetic predisposition in modulating ligamentous tissue strength, structure, and repair processes. Specifically, variations in certain genes involved in extracellular matrix (ECM) composition and collagen synthesis have been associated with an increased risk of ACL injury [9, 10]. One of these, include the gene coding decorin (*DCN*), a small leucine-rich proteoglycan that plays a critical role in collagen fibrillogenesis, ECM organization, and ligament integrity [11–14].

It has been hypothesised that decorin protein binds to collagen fibers and hereby regulates fibril formation, and potentially impacts ligament strength and its ability to withstand mechanical forces. Genetic variations in this key gene may, therefore, contribute to differences in ACL injury risk among individuals. Previous research have identified single nucleotide polymorphisms (SNPs) in the *DCN* gene such as rs13312816 T > A and rs516115 A > G to influence the susceptibility to ACL injuries. Both, rs13312816 T > A and rs516115 A > G, represent intronic variants [15]. Mannion et al. [16] speculated that the haplotype spanning the genomic interval containing rs13312816 T > A and rs516115 A > G, may influence ACL injury susceptibility by having an effect on fibrillogenesis. In addition, Willard et al. [11] reported that rs516115 A > G alleles in combination with alleles from other specific genes encoding proteins implicated in fibrillogenesis to modulate susceptibility to ACL injury.

In this study, we aimed to investigate the association between the two *DCN* SNPs (rs13312816 T > A and rs516115 A > G) and ACL injury risk by comparing a cohort of individuals with ACL injuries to a control group of healthy subjects. Furthermore, within the injured group, we have assessed the relationship between these SNPs and ACL injury-related outcomes, including single and multiple injuries, ACL strain, and the severity of the injury, classified as partial or complete rupture.

## MATERIALS AND METHODS

### Participants

A total of 296 physically active, unrelated, self-reported Caucasian participants were recruited for this case–control genetic association study between the 2017 and 2024. These participants consisted of 160 (male) individuals with ACL injuries (ACLS – ACL strain, ACLRP – ACL partial rupture, ACLRC – ACL rupture complete) and 136 (male) apparently healthy participants without any history of ACL injuries (CON group). All 296 participants from the ACL injury group sustained their injury through non-contact mechanisms.

The participants were soccer players from the Polish 1^st^, 2^nd^ and 3rd division soccer league (trained 11–14 hours per week). The control group were healthy, mainly soccer players, who self-reported no history of ligament or tendon injury. All the male participants (ACL injured and CON groups) were from the same soccer teams, of the same ethnicity (as self-reported, all Polish, East-Europeans for ≥ 3 generations) and had a comparable level of exposure to risk of ACL injury (same volume and intensity of training and match play).

### Ethics committee

The study was approved by the Gdańsk University of Physical Education Ethics Committee (Uchwała nr 1/2024) and written informed consent was obtained from each participant according to the declaration of Helsinki.

### Genetic Analyses

Genomic DNA was extracted from the buccal cells collected from each participants using a GenElute Mammalian Genomic DNA Miniprep Kit (Sigma, Steinheim, Germany) in accordance with the manufacturer’s guidance. Genotyping was conducted in duplicate using TaqMan® Pre-Designed SNP Genotyping Assays (Applied Biosystems, Waltham, MA, USA) on a CFX96 Touch Real-Time PCR Detection System (BIO-RAD, Feldkirchen, Germany) according to the producer’s recommendations. To differentiate the rs13312816 T > A and rs516115 A > G *DCN* variants, the allelic discrimination assays ID: C__32613401_10 and C___2309580_10 contained primers and fluorescently-labeled (FAM and VIC) probes were applicated

### Statistical analysis

The association between the two *DCN* SNPs—rs13312816 T > A (NM_001920.3.-34+1225A > T) and rs516115 A > G (N M_001920.3.324+1090A > G) was assessed both in relation to case-control status and within the case cohort for ACL injury-related outcomes. The analysis was divided into two parts: a primary analysis consisting of a case-control study comparing cases and controls, and a secondary analysis focusing on the cases only. The ACL injury-related outcomes evaluated within the case cohort included ACLF, ACLS, ACLRP, and ACLRC.

Both the primary and secondary analyses involved a single-locus analysis and a haplotype-based analysis. The single-locus analysis was conducted using the R SNPassoc package (version 2.1-0), applying five different genetic models: codominant, dominant, recessive, overdominant, and log-additive. These models were constructed to evaluate the effect of the minor allele on ACL injury outcomes in different ways: the codominant model assesses the effects of having one or two copies of the minor allele compared to having none, the dominant model assumes that having one or two copies of the minor allele is equivalent and compares it to having no copies, the recessive model tests whether the effect is only observed in individuals with two copies of the minor allele, the overdominant model evaluates whether heterozygotes (one copy of each allele) have a different risk compared to both homozygotes and the log-additive model assumes that each additional copy of the minor allele linearly increases the risk. In these models, the minor allele was assumed to be the risk factor. Odds ratios (OR) with 95% confidence intervals (CI) were calculated for each SNP under each genetic model. In all analyses, adjustments were made for potential confounding variables, specifically age and body mass.

The haplotype-based analysis was conducted using the R haplo. stats library (version 1.9.7). Haplotypes were inferred using the expectation-maximization (EM) algorithm, which accounted for phase uncertainty in the haplotype reconstruction. The association between haplotypes and ACL injury outcomes was then assessed using a generalized linear model (GLM) framework, which incorporated the uncertainty from haplotype inference (*haplo.glm* function from the haplo.stats library). The most common haplotype served as the baseline (reference) for comparison. As with the single-locus analysis, age and body mass were included as covariates to adjust for potential confounding effects.

Comparisons of demographic variables between cases and controls were performed using Wilcoxon test for continuous variables and Fisher’s exact test for categorical variables. A P-value < 0.05 was considered statistically significant across all analyses.

## RESULTS

Characteristics of the cases and controls are presented in Table 1. In the primary case-control analysis, we compared the genotype frequencies of rs13312816 T > A and rs516115 A > G between the cases and controls. To account for significant demographic differences, the analysis was adjusted for age (27 ± 4 years for controls vs. 36 ± 7 years for cases, P < 0.001) and body mass (73 ± 16 kg for controls vs. 78 ± 10 kg for cases, P < 0.001). These adjustments were made to control for the potential confounding effects of age and body mass, as these variables differed significantly between the groups (Table 1). By adjusting for these factors, we aimed to isolate the genetic effects of the two SNPs on the casecontrol status.

**TABLE 1:** Group characteristics

Characteristic	Controls, N = 136^1^	Cases, N = 160^1^	P^2^
Age (years)	27 (4)	36 (7)	< 0.001
Height (cm)	178 (7)	178 (6)	0.284
Missing	0	1	
Body mass (kg)	73 (16)	78 (10)	< 0.001
Missing	0	1	

**rs13312816**			0.004
AT	8 / 136 (5.9%)	27 / 158 (17%)	
TT	128 / 136 (94%)	131 / 158 (83%)	
Missing	0	2	

**rs516115**			0.118
AA	85 / 136 (63%)	82 / 159 (52%)	
AG	40 / 136 (29%)	65 / 159 (41%)	
GG	11 / 136 (8.1%)	12 / 159 (7.5%)	
Missing	0	1	

**ACLF**			NA
No	NA	101 / 160 (63%)	
Yes	NA	59 / 160 (37%)	

**ACLS**			NA
No	NA	31 / 160 (19%)	
Yes	NA	129 / 160 (81%)	

**ACLRP**			NA
No	NA	120 / 160 (75%)	
Yes	NA	40 / 160 (25%)	

**ACLRC**			NA
No	NA	141 / 160 (88%)	
Yes	NA	19 / 160 (12%)	

1 n / N (%) for categorical variables, mean (SD) for continuous variables,

2 Wilcoxon rank sum test; Fisher’s exact test, ACLF – ACL full rupture, ACLS – ACL strain, ACLRP – ACL partial rupture, ACLRC – ACL rupture complete, ACL – anterior cruciate ligament, NA – not applicable

The allelic distribution for SNPs rs13312816 T > A and rs516115 A > G between ACL cases and controls is summarized in Table 2.

**TABLE 2:** The allele frequency distributions for *DCN* rs13312816 T > A and *DCN* rs516115 A > G between controls and cases, together with the Hardy-Weinberg equilibrium (HWE) p-values, and odds ratios (OR)

SNP		Control (%)	Cases (%)	Pooled (%)	P^1^
rs13312816	n	272	316	588	0.0047 3.08 (1.33–7.98)
	A	8 (2.94)	27 (8.54)	35 (5.95)	
	T	264 (97.06)	289 (91.46)	553 (94.05)	
	HWE P^2^	1.0	0.605	0.610	

rs516115	n	272	318	590	0.157 1.31 (0.89–1.95)
	G	62 (22.79)	89 (27.99)	151 (25.59)	
	A	210 (77.21)	229 (72.01)	439 (74.41)	
	HWE P^2^	0.084	1.0	0.284	

1 P-value for comparison between controls and cases, with odds ratio (OR) and 95% confidence interval (CI),

2 HWE P-value for genotype frequencies compared to the expected theoretical distribution.

The minor A allele for rs13312816 T > A, was significantly more common in cases (8.54%) compared to controls (2.94%), with a pooled frequency of 5.95% (P = 0.0047, OR = 3.08; 95% CI: 1.33–7.98). Although the G allele of rs516115 A > G was more frequent in cases (27.99%) compared to controls (22.79%), this difference did not reach statistical significance (P = 0.157). No statistically significant deviations for HWE were noted for either of the SNPs (P > 0.05) (Table 2). No A/A genotypes were observed for rs13312816 T > A, the codominant model showed that the T/T genotype was predominant among controls, while the A/T genotype was significantly more frequent in cases (A/T vs T/T: Padj = 0.008; OR = 3.3; 95% CI = 1.44–7.53) (Table 3). No significant differences in genotype distribution were noted for rs516115 (A > G) in any of the analyzed genetic models (Table 4).

**Table 3:** The genotype distributions for rs13312816 T > A between the control and case groups under a codominant model

Model	Controls (%)	Cases (%)	OR (95% CI)	P	Padj
Codominant					
	n=136	n=158			
T/T	128 (94.1)	131 (82.9)	1.0	0.0023	0.008
A/T	8 (5.9)	27 (17.1)	3.3 (1.44–7.53)		

Padj – model including age and body mass, The analysis presented in the table focuses solely on a codominant model due to the absence of individuals homozygous for the A allele (A/A genotype).

**Table 4:** Association of rs516115 A > G genotypes with case-control status under 5 different genetic models

Model	Controls (%)	Cases (%)	OR (95% CI)	P	Padj
Codominant					
A/A	85 (62.5)	82 (51.6)	1.00	0.116	0.281
A/G	40 (29.4)	65 (40.9)	1.68 (1.02–2.77)		
G/G	11 (8.1)	12 (7.5)	1.13 (0.47–2.71)		

Dominant					
A/A	85 (62.5)	82 (51.6)	1.00	0.059	0.359
A/G-G/G	51 (37.5)	77 (48.4)	1.57 (0.98–2.49)		

Recessive					
A/A-A/G	125 (91.9)	147 (92.5)	1.00	0.863	0.352
G/G	11 (8.1)	12 (7.5)	0.93 (0.40–2.18)		

Overdominant					
A/A-G/G	96 (70.6)	94 (59.1)	1.00	0.040	0.141
A/G	40 (29.4)	65 (40.9)	1.66 (1.02–2.70)		

log-Additive					
0,1,2	136 (46.1)	159 (53.9)	1.30 (0.90–1.87)	0.161	0.751

Padj – model including age and body mass

rs13312816 T > A and rs516115 A > G inferred haplotype analyses showed that the TA haplotype was the most prevalent (74.4%) while the AG haplotype was relative rare (< 6.0%) in the study participants. It was interesting to note that the AA haplotype was absent. A regression model using *DCN* haplotypes was employed to explore the association between different haplotypes and casecontrol status (Table 5). The [A;G] haplotype showed a significant positive effect, with a coefficient of 1.19 (standard error, SE = 0.4, P = 0.0047, Padj = 0.0095) and an OR of 3.29 (95% CI: 1.44–7.52), suggesting that individuals with the [A;G] haplotype had more than three times higher odds of being a case compared to those with the reference (baseline) [T;A] haplotype. Conversely, the [T;G] haplotype showed a negative coefficient of -0.02, but this was not statistically significant (P = 0.927).

**Table 5:** The inferred *DCN* (rs13312816 T > A, rs516115 A > G) haplotype association analysis

Term	Coef.	SE	P	Padj
(Intercept)	0.044	0.15	0.7712	< 0.0001
[A;G]	1.19	0.425	0.0047	0.0095
[T;G]	-0.02	0.2155	0.927	0.340

Padj age and body mass adjusted

## Evaluating the potential relationship between the two DCN SNPs on injury severity

In addition to the case-control comparison, we conducted a secondary analysis focusing on the case group only, evaluating the relationship between the two SNPs and ACL injury-related variables. The distribution of ACL injury-related outcomes within the case cohort is summarized in Table 1. The results of the association analysis of rs13312816 T > A with ACL injury-related outcomes are presented in Table 6.

**Table 6:** The evaluation of the rs13312816 T > A genotype distribution frequencies with single and multiple ACL injury related outcomes

Outcome	Model		0 (%)	1 (%)	OR (95% CI)	P	Padj
ACLF	COD	T/T	90 (89.1)	41 (71.9)	1.00	0.007	0.010
(0 – Single, 1 – Multiple)		A/T	11 (10.9)	16 (28.1)	3.19 (1.36–7.48)		

ACLS	COD	T/T	27 (90.0)	104 (81.2)	1.00	0.227	0.205
(0 – No, 1 – Yes)		A/T	3 (10.0)	24 (18.8)	2.08 (0.58–7.42)		

ACLRP	COD	T/T	97 (81.5)	34 (87.2)	1.00	0.403	0.354
(0 – No, 1 – Yes)		A/T	22 (18.5)	5 (12.8)	0.65 (0.23–1.85)		

ACLRC	COD	T/T	114 (82.0)	17 (89.5)	1.00	0.393	0.400
(0 – No, 1 – Yes)		A/T	25 (18.0)	2 (10.5)	0.54 (0.12–2.47)		

ACLF ACL injury frequency – single vs multiple, ACLS ACL strain, ACLRRP – ACL partial rupture, ACLRC ACL complete rupture, COD – codominant model, Padj – adjusted for age and body mass

For rs13312816 T > A, only the codominant model was analyzed due to the absence of A/A homozygotes in the study population. A significant association was observed only for the ACLF outcome (single *versus* multiple), where the A/T genotype was more frequent in cases (28.1%) compared to controls (10.9%) (P = 0.007, Padj = 0.010; OR = 3.19; 95% CI: 1.36–7.48). No further associations were observed for the other ACL injury-related outcomes. Specifically, for ACLS, ACLRP, and ACLRC, no significant associations were identified. Although the A/T genotype was slightly more frequent in cases for ACLS (18.8%) and ACLRP (12.8%), the associations were not statistically significant (P > 0.05 for all models).

The results of the single-locus association analysis of rs516115 A > G with ACL injury-related outcomes are presented in Table 7. A significant association was observed only for the ACLF outcome (single *versus* multiple). The A/G and G/G genotypes were significantly associated with ACLF across all the genetic models. After adjusting for age and body mass, almost all associations remained significant, except for the recessive model, where the association became marginal. In contrast, no significant associations were found for other ACL injury-related outcomes, such as ACLS, ACLRP, and ACLRC.

**Table 7:** The evaluation of the rs516115 A > G genotype distribution frequencies with ACL injury related outcomes

Outcome	Model		0 (%)	1 (%)	OR (95% CI)	P	Padj
ACLF (0 – Single, 1 – Multiple)	COD	A/A	68 (67.3)	14 (24.1)	1.00	< 0.0001	< 0.0001
		A/G	29 (28.7)	36 (62.1)	6.03 (2.83–12.83)		
		G/G	4 (4.0)	8 (13.8)	9.71 (2.57–36.77)		

	DOM	A/A	68 (67.3)	14 (24.1)	1.00	< 0.0001	< 0.0001
		A/G-G/G	33 (32.7)	44 (75.9)	6.48 (3.12–13.45)		

	REC	A/A-A/G	97 (96.0)	50 (86.2)	1.00	0.027	0.060
		G/G	4 (4.0)	8 (13.8)	3.88 (1.11–13.51)		

	HET	A/A-G/G	72 (71.3)	22 (37.9)	1.00	< 0.0001	< 0.0001
		A/G	29 (28.7)	36 (62.1)	4.06 (2.05–8.05)		

	ADD	0,1,2	101 (63.5)	58 (36.5)	4.24 (2.33–7.71)	< 0.0001	< 0.0001

ACLS (0 – No, 1 – Yes)	COD	A/A	17 (54.8)	65 (50.8)	1.00	0.536	0.564
		A/G	13 (41.9)	52 (40.6)	1.05 (0.47–2.35)		
		G/G	1 (3.2)	11 (8.6)	2.88 (0.35–23.84)

	DOM	A/A	17 (54.8)	65 (50.8)	1.00	0.685	0.576
		A/G-G/G	14 (45.2)	63 (49.2)	1.18 (0.54–2.59)		

	REC	A/A-A/G	30 (96.8)	117 (91.4)	1.00	0.267	0.303
		G/G	1 (3.2)	11 (8.6)	2.82 (0.35–22.69)		

	HET	A/A-G/G	18 (58.1)	76 (59.4)	1.00	0.894	0.960
		A/G	13 (41.9)	52 (40.6)	0.95 (0.43–2.10)		

	ADD	0,1,2	31 (19.5)	128 (80.5)	1.28 (0.67–2.44)	0.450	0.392

ACLRP (0 – No, 1 – Yes)	COD	A/A	58 (48.3)	24 (61.5)	1.00	0.163	0.336
		A/G	54 (45.0)	11 (28.2)	0.49 (0.22–1.10)		
		G/G	8 (6.7)	4 (10.3)	1.21 (0.33–4.39)		

	DOM	A/A	58 (48.3)	24 (61.5)	1.00	0.150	0.218
		A/G-G/G	62 (51.7)	15 (38.5)	0.58 (0.28–1.22)		

	REC	A/A-A/G	112 (93.3)	35 (89.7)	1.00	0.475	0.737
		G/G	8 (6.7)	4 (10.3)	1.60 (0.45–5.63)		

	HET	A/A-G/G	66 (55.0)	28 (71.8)	1.00	0.060	0.140
		A/G	54 (45.0)	11 (28.2)	0.48 (0.22–1.05)		

	ADD	0,1,2	120 (75.5)	39 (24.5)	0.78 (0.43–1.41)	0.403	0.426

ACLRC (0 – No, 1 – Yes)	COD	A/A	72 (51.4)	10 (52.6)	1.00	0.480	0.222
		A/G	56 (40.0)	9 (47.4)	1.16 (0.44–3.04)		
		G/G	12 (8.6)	0 (0.0)	0.00 (0.00)		

	DOM	A/A	72 (51.4)	10 (52.6)	1.00	0.922	0.908
		A/G-G/G	68 (48.6)	9 (47.4)	0.95 (0.37–2.49)		

	REC	A/A-A/G	128 (91.4)	19 (100.0)	1.00	0.363	0.086
		G/G	12 (8.6)	0 (0.0)	0.00 (0.00)		

	HET	A/A-G/G	84 (60.0)	10 (52.6)	1.00	0.542	0.566
		A/G	56 (40.0)	9 (47.4)	1.35 (0.52–3.53)		

	ADD	0,1,2	140 (88.1)	19 (11.9)	0.77 (0.35–1.72)	0.480	0.521

ACLF ACL injury frequency – single vs multiple, ACLS ACL strain, ACLRRP – ACL partial rupture, ACLRC ACL complete rupture; COD – codominant model, DOM – dominant model, REC – recessive model, HET – over-dominant heterozygote model, ADD – logadditive model, Padj – P value adjusted for age and body mass

Specifically, in the analysis of ACLF, a significant association was observed between the A/G and G/G genotypes in the codominant model compared to the reference A/A genotype. The A/G genotype was significantly more frequent in cases (62.1%) than controls (28.7%), with an OR of 6.03 (95% CI: 2.83–12.83, P < 0.0001, Padj < 0.0001). Similarly, the G/G genotype also showed a higher prevalence in cases (13.8%) compared to controls (4.0%), with an OR of 9.71 (95% CI: 2.57–36.77, P < 0.0001, Padj < 0.0001). In the dominant model (A/G-G/G vs. A/A), the combined A/G-G/G genotypes were significantly associated with ACLF, yielding an OR of 6.48 (95% CI: 3.12–13.45, P < 0.0001, Padj < 0.0001). The recessive model also demonstrated a significant association with an OR of 3.88 (95% CI: 1.11–13.51, P = 0.027, Padj = 0.060). For the overdominant model (HET), the A/G genotype was more prevalent in cases (62.1%) compared to controls (28.7%), showing an OR of 4.06 (95% CI: 2.05–8.05, P < 0.0001, Padj < 0.0001). The log-additive model further supported this finding with an OR of 4.24 (95% CI: 2.33–7.71, P < 0.0001, Padj < 0.0001).

In the haplotype-based analysis (Table 8), significant associations were observed once again only for the ACLF outcome.

**Table 8:** The *DCN* rs13312816 T > A and rs516115 A > G inferred haplotype association analyses for haplotype-based association analysis for the ACL injury related outcomes in the cases cohort

Outcome	Term	coef.	SE	P	Padj
ACLF	(Intercept)	-1.43	0.27	< 0.0001	0.0098
	[A;G]	1.21	0.47	0.010	0.0099
	[T;G]	1.57	0.36	< 0.0001	< 0.0001

ACLS	(Intercept)	1.32	0.26	< 0.0001	0.781
	[A;G]	0.68	0.65	0.292	0.271
	[T;G]	0.02	0.42	0.9593	0.878

ACLRP	(Intercept)	-0.97	0.24	< 0.0001	0.016
	[A;G]	-0.38	0.53	0.478	0.463
	[T;G]	-0.19	0.39	0.627	0.649

ACLRC	(Intercept)	-1.89	0.32	< 0.0001	0.960
	[A;G]	-0.62	0.78	0.430	0.425
	[T;G]	-0.08	0.51	0.881	0.874

ACLF ACL injury frequency – single vs multiple, ACLS ACL strain, ACLRRP – ACL partial rupture, ACLRC ACL complete rupture, Padj – adjusted for age and body mass, coef. – regression coefficient, SE – standard error

The [A;G] haplotype reflected a significant association with an odds ratio equivalent to a coef = 1.21 (SE = 0.47, P = 0.01, Padj = 0.0099). Additionally, the [T;G] haplotype demonstrated an even stronger association, with a coef = 1.57 (SE = 0.36, P < 0.0001, Padj < 0.0001). For the other ACL injury-related outcomes, no significant associations were identified. In the analysis of ACLS, neither [A;G] (P = 0.292, Padj = 0.271) nor [T;G] (P = 0.9593, Padj = 0.878) showed any significant association with the outcome. Similarly, for ACLRP and ACLRC, no significant associations were found for [A;G] or [T;G] (Padj > 0.05 for all models).

**FIG. 1:**
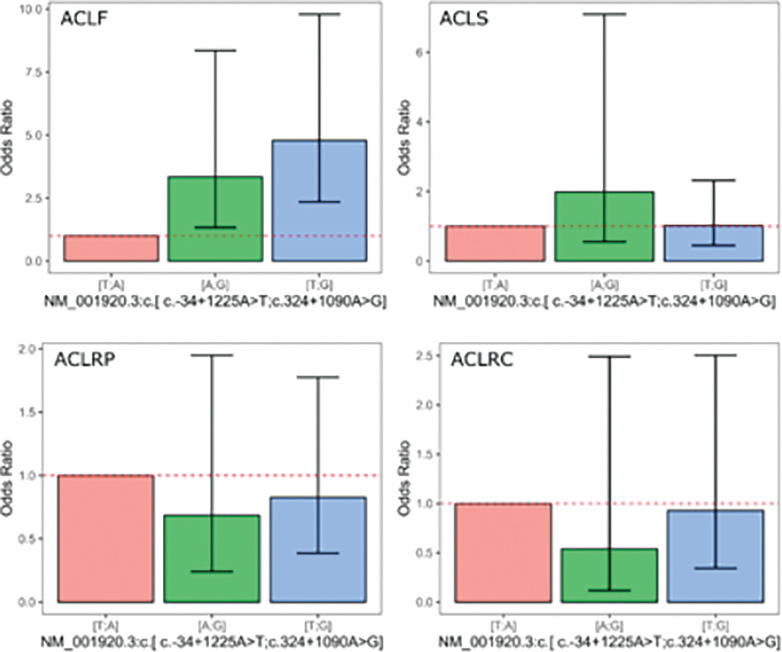
Odds ratios with 95% confidence intervals from the regression models.

Figure 1 presents the plots for all outcomes, displaying the ORand 95% confidence intervals derived from the coefficients for the genetic models based on the *DCN* rs13312816 T > A and rs516115 A > G inferred haplotype. The [A;G] inferred haplotype was associated with a substantial increase in the odds of multiple ACL injuries, with an OR of 3.34 (95% CI: 1.33–8.35).

## DISCUSSION

Our study found that individuals who had inherited the A allele of *DCN* rs13312816 T > A had a threefold higher susceptibility (OR = 3.08) compared to non-carriers. Notably, the A/T genotype was overrepresented in cases, and associated with a threefold increase susceptibility compared non carriers (OR = 3.19). Those individuals carrying the A/T genotype have a higher risk of developing multiple ACL injuries compared to those with the T/T genotype. Emphasizing the A allele’s potential impact on reduced ligament resilience and impairment of the biomechanical integrity of the ACL. No associations were noted for rs516115 A/G in our study cohort. Our findings align with Candela et al. [17], who highlighted the complex interplay of genetic variants in structural proteins like decorin, type 1 collagen, and biglycan in ACL integrity. Candela et al. [17] identified both *DCN* rs516115 and rs13312816 as relevant, albeit inconclusive as noted from the variability in outcomes across studies, and suggested the need for more robust research on *DCN*’s influence on ligament resilience. Kaynak et al. [18] also reviewed these SNPs, finding that while proteoglycan genes (including *DCN*) are critical in ACL structure, results across studies remain inconsistent. These reviews support our findings by acknowledging that while *DCN* rs13312816 may impact ACL resilience, associations with rs516115 are less definitive, reinforcing the genetic complexity underlying ACL injuries.

The haplotype analyses did however implicate a genomic interval spanning both the *DCN* rs13312816 T > A and rs516115 A > G SNPs. More specifically, the [A;G] haplotype of NM_001920.3 significantly increased the risk of ACL injury recurrence (OR = 3.29), particularly for multiple injuries, while the [T;G] haplotype exhibited an even stronger association with recurrent injuries (OR = 4.79, 95% CI: 2.35–9.79) suggesting that the odds of multiple ACL injuries in individuals with this haplotype were nearly five times higher than in those with the baseline haplotype. Individuals carrying the [A;G] haplotype had more than three times higher odds of experiencing multiple ACL injuries compared to those with the reference haplotype [T;A]. These findings suggest that specific *DCN* haplotypes may exacerbate ligament susceptibility to repetitive strain, possibly by influencing collagen organization and stability [19]. These results are in line with Mannion et al. [16], who identified significant associations between proteoglycan gene haplotypes (including *DCN* and *BGN*) and ACL injury susceptibility. Their study also noted sex-specific effects, with *DCN* and *ACAN* (Aggrecan gene) variants being significant among female participants, suggesting that genetic impacts on ligament resilience may vary based on sex. Our findings on recurrent injury risk align with Cięszczyk et al. [20], who found that *BGN* haplotypes impacted ACL rupture risk differently in males and females, indicating that haplotype configurations, aligning with specific genetic sequencing motifs, may interact with biological factors to influence ligament stability. Together, these studies highlight the potential of haplotype-based analyses in identifying high-risk individuals, especially for recurrent injuries.

Our study’s focus on *DCN* aligns with findings from Suijkerbuijk et al. [21], who explored the impact of genetic polymorphisms in inflammatory and ECM pathways on ACL integrity. By examining *IL1B* and *IL6* polymorphisms in conjunction with ECM components like *DCN* and *BGN*, Suijkerbuijk et al. proposed that these genes modulate ECM remodeling, impacted ACL susceptibility. This study underscores the broader context in which *DCN* variations may contribute to ACL injury risk by affecting the inflammatory and remodeling response post-injury. Our findings that specific *DCN* haplotypes are associated with re-injury risk, may reflect decorin’s role in balancing collagen synthesis and ECM remodeling during the recovery process. Willard et al. [11] further expanded this view by studying miRNA binding motifs associated with ECM genes like *DCN, COL5A1*, and *BGN*, identifying miRNAs that regulate these genes post-transcriptionally. They found sex-specific allele combinations associated with ACL risk, suggesting that miRNA-mediated regulation of *DCN* may impact ACL susceptibility. Given that miRNAs affect ECM gene expression and, consequently, ligament structure, our findings on *DCN* rs13312816 and associated haplotypes might be influenced by miRNA interactions, which could affect collagen fibrillogenesis and contribute to the observed injury susceptibility [16].

It becomes evident that decorin plays an essential, phase-specific role in tendon and ligament integrity by modulating collagen fibril organization, influencing ECM remodeling, and working in tandem with other proteoglycans like biglycan. In Abbah et al. [22], decorin was studied in the context of tendon fibrosis, where it was shown to mitigate excessive ECM deposition by inhibiting TGF-β1, and cytokine known to drive fibrosis in injured tendons. This study’s co-transfection of *DCN* and *IL-10* genes in human tenocytes showed a synergistic effect in reducing TGF-β1-induced ECM remodeling. In our findings, the relevance of decorin’s anti-fibrotic properties is highlighted, as genetic variants like rs13312816 could influence decorin’s features and thereby impact ECM homeostasis within the ACL. The use of a collagen hydrogel for sustained release of decorin and IL-10 plasmids suggests that decorin’s therapeutic potential may be best realized in a controlled-release system, which could be explored for ACL repair in genetically predisposed individuals [22]. This aligns with our study’s proposal that decorin’s influence on collagen stability could in the future be a therapeutic target for those with *DCN* haplotypes linked to ACL susceptibility. Beach et al. [23] demonstrated that decorin knockdown enhances tendon stiffness in aged, but not geriatric, mice. Decorin’s influence on collagen fibril diameter was evident in this study, where knockdown in aged tendons increased fibril heterogeneity—a change associated with increased resilience and modulus. However, in older tendons, these benefits were not sustained, implying that decorin’s effects are age-dependent. *DCN* variants and ACL susceptibility could similarly reflect age-related nuances in decorin function. In young, active individuals at high risk for ACL injuries, decorin’s role in fibril organization might be more significant, similar to the role of collagen [24]. Thus, genetic screening for *DCN* variants in younger populations could inform targeted interventions that leverage decorin’s impact on fibril structure before age-related changes reduce its efficacy. Leahy et al. [25] explored decorin and biglycan in tendon repair phases using a TMinducible knockout model, revealing that decorin’s role becomes more prominent during the remodeling phase. This temporal specificity is crucial for therapeutic timing: decorin seems to be essential when collagen realignment and ECM stabilization occur in the final healing stages. that variants like rs13312816 could disrupt decorin’s late-phase activity, potentially hindering proper remodeling after initial ACL injury. The insights from Leahy et al. indicate that optimizing decorin levels post-ACL repair could support collagen maturation, reducing recurrence risk. Additionally, the phased approach in this study suggests decorin could be targeted through temporally specific gene therapies or controlled-release systems post-injury to enhance ligament repair outcomes. Dunkman et al. [26] observed that decorin is crucial for later-stage tendon healing, particularly in collagen fibril fusion and maturation. Decorin-null tendons displayed smaller fibril diameters post-injury, suggesting that decorin facilitates collagen fibrillogenesis and maintains proper fibril dimensions.

This study investigated the role of decorin (*DCN*), albeit DCN together with other proteoglycans, participate in ligament healing, with a specific focus on ACL injuries [27]. DCN is involved in collagen fibril maturation, and genetic variations in DCN expression may impact the healing process, making ligaments more susceptible to reinjury. A decorin deficiency, whether genetic or due to other factors, could hinder collagen alignment, weakening the ligament and increasing the risk of subsequent damage. Research suggests that targeting both decorin and biglycan during ACL repair could offer a more comprehensive approach, supporting healing at different stages. Findings from studies indicate that a balanced level of decorin, rather than its complete suppression, is optimal for collagen organization, leading to stronger and more aligned fibrils, as seen in the “MIX group” from Lu et al.’s [28] research. This approach may be especially beneficial for individuals with *DCN* genetic variants that affect decorin levels. The study also highlights the potential of using genetic markers, such as the rs13312816 polymorphism, to identify individuals at higher risk for ACL injuries, allowing for more personalized preventative strategies. By integrating genetic data with environmental and biomechanical factors, such as training intensity and joint laxity, sports medicine professionals could better predict ACL injury risk and tailor interventions to each athlete [29, 30]. This multi-faceted approach could enhance the precision of risk assessments and help manage training loads, strength conditioning, and movement patterns [31]. Moreover, incorporating genetic screening into post-recovery plans could improve long-term prevention strategies and reduce the likelihood of re-injury [10]. Genetic testing may become a key component of sports medicine, enabling early identification of high-risk individuals and supporting targeted, data-driven interventions [17]. Further research is needed to refine these genetic markers and evaluate their combined impact with biomechanical data, ultimately leading to personalized ACL injury prevention protocols.

In conclusion, this study provides evidence that specific polymorphisms in the *DCN* gene, particularly rs13312816 and distinct haplotypes such as [A;G] and [T;G], are significantly associated with susceptibility to ACL injuries and, notably, with recurrent injury events. The minor allele A of rs13312816, along with the [A;G] and [T;G] haplotypes, were linked to an increased risk of multiple ACL injuries, suggesting that these genetic variants may compromise ligament resilience by altering collagen organization and stability. Our findings align with the known biological role of decorin in collagen fibrillogenesis, supporting the hypothesis that *DCN* gene variations could disrupt collagen integrity, making ligaments more vulnerable to repetitive mechanical stress. Importantly, this study underscores the value of exploring specific genotypes and haplotypes rather than single polymorphisms to better understand genetic susceptibility to ACL injuries. These insights open possibility for personalized injury prevention strategies. Genetic screening for high-risk alleles, such as rs13312816, could enable targeted interventions, such as personalized training and rehabilitation protocols for individuals at heightened risk of ACL injury or re-injury. In high-risk populations, genetic profiling may eventually become part of a multifactorial risk assessment, integrating biomechanical, environmental, and genetic factors to inform individualized injury prevention and recovery programs. Future research should focus on expanding these findings in larger, diverse cohorts to validate the associations across populations. Additionally, functional studies are needed to elucidate how *DCN* polymorphisms impact collagen structure and ligament biomechanics, which could inform therapeutic strategies for reinforcing ligament resilience. By furthering our understanding of the genetic factors contributing to ACL injuries, this research contributes to the development of more effective, personalized approaches in sports medicine and injury prevention. While advancements have been made in uncovering the genetic contributors to both acute and chronic musculoskeletal soft tissue injuries, notable obstacles continue to slow the advancement of genetics-focused studies. A key limitation in the present association analysis is the modest sample size of individuals who have undergone ACL injury, especially those with injuries arising from non-contact events. Expanding the participant pool to include a greater number of ACL injury cases is critical to overcoming this issue. Furthermore, because the current findings do not offer compelling support for a connection between *DCN* gene variants and ACL rupture susceptibility, upcoming research should expand the scope of investigation beyond the two SNPs currently studied in this gene in our study. The development of high-throughput sequencing technologies, enhanced genotyping platforms, and powerful bioinformatics pipelines now makes this kind of in-depth exploration practical. In addition, any novel variants uncovered through next-generation sequencing approaches should be tested across several independent populations to validate their association and establish them as dependable genetic markers of risk.

## CONCLUSIONS

We found significant differences in the genotype and allele frequencies between ACL cases and controls for rs13312816 T > A, with the A/T genotype showing a notably higher prevalence in cases, particularly in individuals with multiple ACL injuries. The association between the A/T genotype and increased incidence of injury highlights the potential importance of this genetic variation in predisposition to anterior cruciate ligament injuries. On the other hand, the rs516115 A > G SNP did not exhibit significant associations with ACL injury, although a trend was observed for its involvement in ACL injury outcomes, such as ACL strain and partial rupture. Additionally, haplotype analysis revealed that the [A;G] haplotype is associated with a higher risk of ACL injury, emphasizing the importance of considering multiple SNPs together rather than severally. These findings highlight the role of genetic variations in influencing injury risk, suggesting that SNPs in the *DCN* gene could contribute to the development of ACL injuries. Future research should continue to explore the mechanisms underlying these genetic effects, incorporating larger and more diverse cohorts, to better understand the genetic factors influencing ACL injury and the potential for personalized injury prevention strategies.
